# Severe Exercise-Induced Laryngeal Obstruction Treated With Supraglottoplasty

**DOI:** 10.3389/fsurg.2019.00044

**Published:** 2019-07-31

**Authors:** Astrid Sandnes, Magnus Hilland, Maria Vollsæter, Tiina Andersen, Ingvild Øvstebø Engesæter, Lorentz Sandvik, John-Helge Heimdal, Thomas Halvorsen, Geir Egil Eide, Ola Drange Røksund, Hege H. Clemm

**Affiliations:** ^1^Department of Internal Medicine, Innlandet Hospital Trust, Gjøvik, Norway; ^2^Department of Clinical Science, University of Bergen, Bergen, Norway; ^3^Department of Otolaryngology/Head and Neck Surgery, Haukeland University Hospital, Bergen, Norway; ^4^Department of Pediatrics, Haukeland University Hospital, Bergen, Norway; ^5^Norwegian Advisory Unit on Home Mechanical Ventilation, Thoracic Department, Haukeland University Hospital, Bergen, Norway; ^6^Department of Physiotherapy, Haukeland University Hospital, Bergen, Norway; ^7^Department of Surgery, Haukeland University Hospital, Bergen, Norway; ^8^Department of Clinical Medicine, University of Bergen, Bergen, Norway; ^9^Centre for Clinical Research, Haukeland University Hospital, Bergen, Norway; ^10^Department of Global Public Health and Primary Care, University of Bergen, Bergen, Norway; ^11^The Faculty of Health and Social Sciences, Western Norway University of Applied Sciences, Bergen, Norway

**Keywords:** EILO, VCD, supraglottoplasty, larynx, exercise induced laryngeal obstruction

## Abstract

**Introduction:** Exercise induced laryngeal obstruction (EILO) is relatively common in adolescents, with symptoms often confused with exercise induced asthma. EILO often starts with medial or inward rotation of supraglottic structures of the larynx, whereas glottic adduction appears as a secondary phenomenon in a majority. Therefore, surgical treatment (supraglottoplasty) is used in thoroughly selected and highly motivated patients with pronounced symptoms and severe supraglottic collapse.

**Aim:** To investigate efficacy and safety of laser supraglottoplasty as treatment for severe supraglottic EILO by retrospective chart reviews.

**Methods:** The EILO register at Haukeland University Hospital, Bergen, Norway was used to identify patients who had undergone laser supraglottoplasty for severe supraglottic EILO, verified by continuous laryngoscopy exercise (CLE) test, during 2013–2015. Laser incision in both aryepiglottic folds anterior to the cuneiform tubercles and removal of the mucosa around the top was performed in general anesthesia. Outcomes were questionnaire based self-reported symptoms, and laryngeal obstruction scored according to a defined scheme during a CLE-test performed before and after surgery.

**Results:** Forty-five of 65 eligible patients, mean age 15.9 years, were included. Post-operatively, 38/45 (84%) patients reported less symptoms, whereas CLE-test scores had improved in all, of whom 16/45 (36%) had no signs of obstruction. Most improvements were at the supraglottic level, but 21/45 (47%) also improved at the glottic level. Two of 65 patients had complications; self-limiting vocal fold paresis and scarring/shortening of plica ary-epiglottica.

**Conclusion:** Supraglottoplasty improves symptoms and decreases laryngeal obstruction in patients with severe supraglottic EILO, and appears safe in highly selected cases.

## Introduction

Exercise induced laryngeal obstruction (EILO) is relatively common in young individuals (1–5). Symptoms are primarily prolonged and/or noisy inspiration and shortness of breath during ongoing high intensity exercise (6). Continuous laryngoscopy exercise test (CLE-test) (7) visualize the progression of obstruction during ongoing exercise from start to exhaustion, being essential for proper diagnosis and for subsequent planning and choice of treatment (8–11). Typically, the larynx appears endoscopically normal at rest whereas a transient, reversible narrowing occurs as exercise intensity increases. EILO is increasingly recognized as an important differential diagnosis to exercise-induced asthma/bronchoconstriction (EIB) in otherwise healthy adolescents (6, 10, 12–15).

Multiple treatment options are being applied for EILO, including surgical and non-surgical approaches, but so far none are based on high-quality evidence (2, 4, 10, 16). The etiology of EILO is likely to be heterogeneous, and at least two distinct phenotypes have been suggested; one characterized predominantly by a supraglottic medialization and/or collapse that clearly precedes the glottic obstruction, and another phenotype in which the glottic obstruction seems to incite the sequence of events (5, 6, 17). Treatment must be individualized and take into account these diverse findings (10). In a clinical setting, the role of surgery should be secondary to conservative treatment, such as properly guided breathing advice, speech therapy or inspiratory muscle training (4, 18–25). The literature suggests that surgery is beneficial in patients with a clearly predominant supraglottic involvement for whom EILO represent a significant clinical problem (4, 10, 11, 26). Follow-up studies for up to 5 years suggest lasting positive results (27, 28).

Surgery in patients with EILO is being performed at several centers, but a literature review revealed that results from only 72 cases have been published (26, 29). In this retrospective study, we investigated the efficacy and safety of laser supraglottoplasty for supraglottic EILO performed at our hospital, with CLE tests performed before and after surgery in all.

## Materials and Methods

### Participants and Study Design

This study was based on a retrospective review of the *EILO-register* at Haukeland University Hospital, Norway. Our unit receives patients with suspected EILO from all Norway, and annually performs ~250 CLE tests. Patients who had been treated surgically with supraglottoplasty for EILO on clinical indications during 2013–2015 were included. First-line therapy for EILO at our institution (received by all patients) was physician-guided structured breathing advice while patients were observing their laryngeal responses on the monitor (biofeedback). Second-line treatment options were speech therapy or physician-guided inspiratory muscle training (IMT), with supraglottoplasty reserved for highly selected supraglottic cases. Indication for supraglottoplasty was based on symptom severity, the extent of the supraglottic collapse during a CLE-test (7), and patient motivation. Patients with laryngomalacia characteristics at rest [omega-shaped/juvenile epiglottis and prominent cuneiform tubercula's and/or redundant mucosa at the arytenoid region (30)], were excluded. Co-existing asthma had been treated according to guidelines (31), and asthma had been excluded as the cause of the patients' symptoms by a clinical interview and if in doubt, by performing spirometry after an exercise test (32). All patients had been informed about the surgical procedure and of risk factors.

The study was approved by the Committee on Medical Research Ethics of Western Norway (REK number 2016/1898), and informed written consent was obtained from the participants and/or their guardian.

### Subjective Symptom Scores

Symptom scores before and after surgery were obtained using a questionnaire that also included demographic background variables. All patients answered these four questions (Q): Q1. “*Have your symptoms improved after the previous test?”, “yes”*/“*no”;* Q2. “*Rate your breathing problem on a scale from 0 to 10*”; Q3. “*I experience inspiratory breathing difficulty when I exercise,”* using a numeric rating scale (NRS) from 1 to 5 (1 = never, 2 = sometimes, 3 = often, 4 = almost every time, 5 = always); Q4. “*When you are physically active, how much are you bothered by your breathing difficulties?”*, using NRS from 1 to 5 (1 = nothing, 2 = a bit, 3 = pretty much, 4 = a lot, and 5 = disabling).

### Spirometry and CLE-Test

Spirometry was performed with a Vmax 22^©^ spirometer (*SensorMedics, Yorba Linda, CA, USA*) according to guidelines (33). A trans-nasal flexible fiberoptic laryngoscope (Olympus ENF-P3^©^, Tokyo, Japan), diameter 3.5 mm, introduced after applying a decongestive nasal spray (Rhinox^©^) and local anesthesia (Xylocaine^©^), was secured using a custom designed helmet in a position allowing for a good view of supraglottic structures and the vocal folds. Maximum voluntary ventilation (MVV) and ergospirometry data were collected in conjunction with the CLE-test using a Jaeger Oxycon Pro Cardiopulmonary Exercise testing system *(Viasys Health Care, Yorba Linda, CA, USA)*. Continuous video-recorded laryngoscopy throughout a maximal cardiopulmonary exercise test on a treadmill *(Woodway ELG 70, Weil am Rhein, Germany)* was performed as previously described (the CLE-test) (7) before and after surgery. Simultaneous a computerized and modified Bruce ramp protocol coupled with integrated video-recording of the upper part of the body and sound-recordings was performed. The treadmill protocol increased speed and/or elevation every 1 min, aiming to reach maximum exercise capacity after 6–14 min (34). The test was considered successful if the patient continued until exhaustion or was stopped by respiratory distress, preferably supported by a plateau in oxygen consumption and/or heart rate response.

### Evaluation of CLE-Test

**Figure 1:**
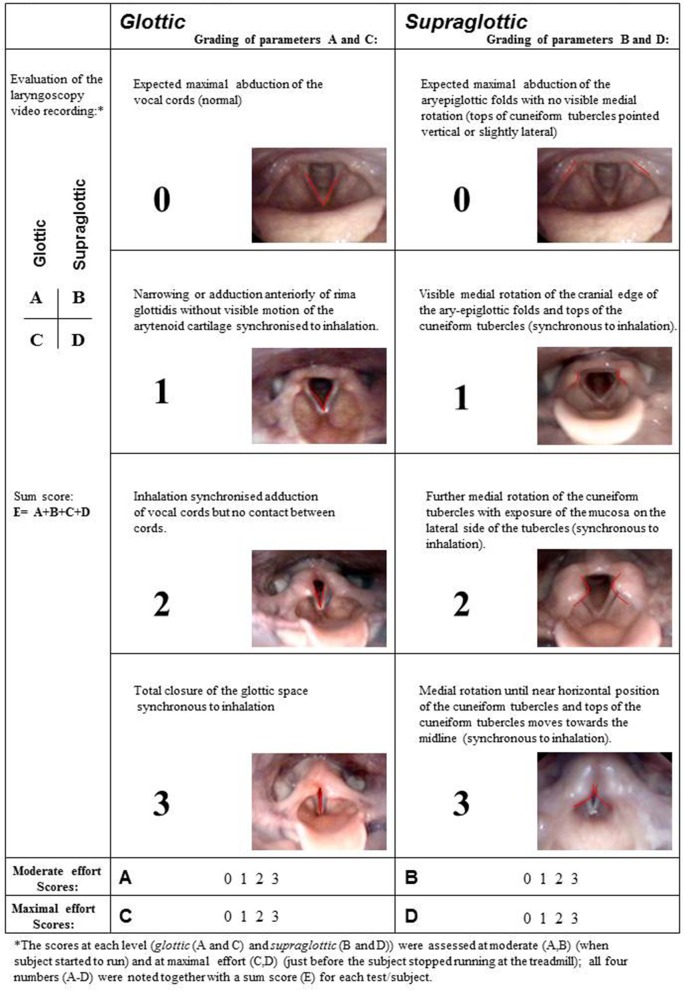
Grading system of laryngeal obstruction according to Maat et al. (35), reproduced with permission.

Laryngeal movements were scored as previously described at moderate and maximum exercise intensity, both at the glottic and supraglottic level (Figure 1) (2, 35). The assessments were done retrospectively, using the stored video-recorded CLE-tests presented in pairs (pre-post) to four experienced raters (HHC, ODR, MH and JHH). A blinded procedure was tested but proved impossible, as the surgical changes were impossible to hide. Thus, assessments were open and consensus-based. A score of ≥2 at either glottic or supraglottic level was interpreted as abnormal. Supraglottic EILO was defined by the supraglottic obstruction preceding the glottic obstruction and supraglottic (D) scores > glottic (C) scores at maximum exercise. Patients referred for supraglottoplasty had a supraglottic score ≥2, except one patient included because of large involvement from a retroflex epiglottis. Obstruction of the vocal folds (i.e., the glottic level) was not an exclusion criterion, but the supraglottic adduction should be the inciting event and clearly the predominant element of the obstruction.

### Supraglottoplasty

**Figure 2:**
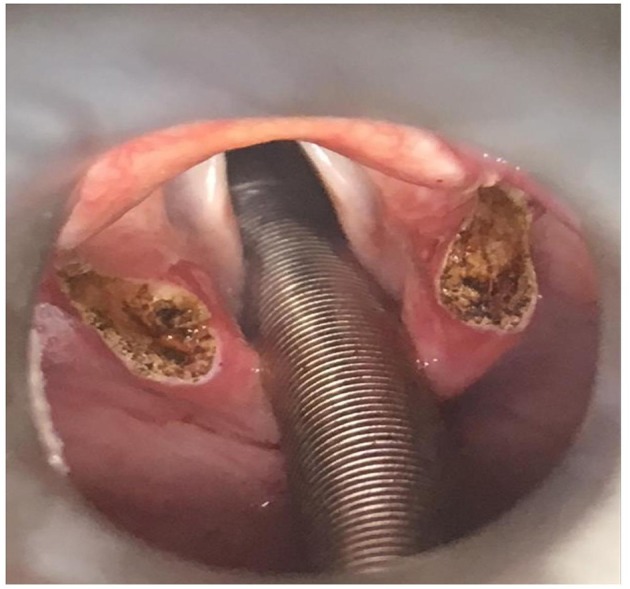
Supraglottoplasty with carbon dioxide laser on a patient with exercise induced laryngeal obstruction (EILO). Laser beams of 2–4 Watt focused with micro spot was utilized. The aryepiglottic fold was split anteriorly down to the level of the musculus aryepiglotticus approaching the cranial margin of plica ventricularis. Then tissue around the top of the cuneiform cartilages was removed in a circular pattern, creating a triangular shaped excision.

All patients underwent surgery in general anesthesia with suspension micro-laryngoscopy and endoscopic supraglottoplasty with carbon dioxide laser, performed by one of two surgeons, experienced with laryngoplasty. The oral laser endotracheal (LET) tube was positioned posteriorly, protecting the interarytenoid area. A Lindholm/Benjamin laryngoscope was introduced into the vallecular exposing both aryepiglottic folds and epiglottis. The arytenoid was grasped with micro laryngeal forceps and pulled slightly forward and medially stretching the aryepiglottic fold, revealing the amount of abundant arytenoid tissue. Laser beams of 2–4 Watt focused with micro spot was utilized. The aryepiglottic fold was split anteriorly down to the level of the musculus aryepiglotticus approaching the cranial margin of plica ventricularis. Then tissue around the top of the cuneiform cartilages was removed in a circular pattern, creating a triangular shaped excision (Figure 2). In cases where the cuneiform tubercles were pointy and exposed in the excision, parts of this cartilage were included. The same procedure was performed bilaterally making sure to avoid endothelial damage to the interarytenoid covered by the LET-tube. In some cases with epiglottic involvement, epiglottotomy and rotation of the epiglottis toward the tongue base (epiglottopexy) were performed. Specific anatomic decisions tailored to the individual patient's anatomy were guided by findings on preoperative video-recording during CLE-test (26, 27, 36).

### Statistical Methods

This was a cross-sectional pre-post study, with main outcomes being CLE-scores and symptom scores obtained before vs. after surgery, compared with Student's paired *t*-test. The CLE-scores are by nature ordinal and categorical, ranging from 0 to 3. Due to the few number of categories, data were calculated and reported as mean values with 95% confidence intervals (CI), as this is considered to provide more information than medians and interquartile ranges (37). To account for multiple correlated measurements in the same test subjects, mixed linear regression with fixed effects including three-way and two-way interactions was applied to address CLE-score changes before vs. after surgery obtained at moderate vs. maximal exercise intensity and at glottic vs. supraglottic laryngeal levels (38). All analyses were performed with SPSS version 24 (SPSS, Chicago, IL, USA).

## Results

Sixty-five patients were eligible during the inclusion period. Twelve patients were excluded due to incomplete datasets, either pre- CLE-test or post-CLE-test, four patients did not consent to participation in the study and four patients were excluded retrospectively due to laryngeal findings characteristic of laryngomalacia. Thus, 45 patients were included; age 10–25 (mean 15.9) years, 14 males and 31 females. Baseline characteristics are outlined in Table 1. The post CLE-test was performed between 4 and 28 months (mean 13.4 months) after surgery. Lung function, distance completed on treadmill, minute ventilation (VE), peak VO_2_ (ml/min) and maximal heart rate did not differ before vs. after surgery. MVV had increased significantly after surgery, and respiratory rate at peak exercise was reduced (Table 2). Epiglottotomy and/or epiglottopexy were performed as additional surgery in five of the 45 patients. As the CLE scoring system does not encompass ways to assess this type of surgery, these outcomes were not tabulated.

**Table 1:** Baseline characteristics of the 45 patients included from the EILO register at Haukeland University Hospital, Bergen (Norway) who were surgically treated for EILO during 2013–2015.

**Variable category**	**Pre-operative**	**Post-operative**	***P*-value^a^**
Female, *n* (% of group)	31/45 (68.9)		
Age in years, mean (range)	15.9 (10–25)	16.6 (11–27)	
Height in cm, mean (SD)	166.2 (10.3)	168.89 (9.4)	<0.001
Weight in kg, mean (SD)	57.7 (11.1)	62.21 (12.2)	<0.001
**LUNGFUNCTION**			
FVC, % of predicted (SD)	104.8 (14.1)	104.3 (14.7)	0.667
FEV_1_, % of predicted (SD)	106.2 (13.1)	104.7 (13.5)	0.285
FEV_1_/FIV_1_ or FEF50/FIF50 > 1.5	2 (4.4)	2 (4.4)	
**TREATMENT BEFORE SURGERY**, ***n*** **(%)**			
First line conservative therapy only	30 (66.7)		
**Second line therapy**			
IMT	14 (31.1)		
Speech therapy	1 (2.2)		
**LEVEL OF LARYNGEAL OBSTRUCTION**, ***n*** **(%)**			
Only supraglottic	4 (9.1)		
Supraglottic ≥2 and glottic = 1	22 (50.0)^*^		
Supraglottic ≥2 and glottic ≥2	18 (40.9)		

*Values are ratios (% of group) or means (SD)*.

a *From Student's paired t-test*.

*EILO, exercise induced laryngeal obstruction; IMT, inspiratory muscle training; FVC, forced vital capacity; FEV_1_, forced expiratory volume in first second time; FEF50, forced expiratory flow at 50%; FIF50, forced inspiratory flow at 50%; SD, standard deviation*.

* *One patient with supraglottic score 1 and glottic score 1 was included because of large involvement from a retroflex epiglottis (not assessed in the scoring system)*.

**Table 2:** Ergospirometry data of the 45 patients included from the EILO register at Haukeland University Hospital, Bergen (Norway) who were surgically treated for EILO during 2013–2015.

**Measures^a^**	**Pre-operative**	**Post-operative**	***p*-value^b^**
Distance on treadmill; m	677 (607, 748)	680 (623, 738)	0.858
Heart rate; per min	184 (180, 189)	177 (165, 189)	0.200
VO_2_ max; ml/min	2760 (2,540, 2,979)	2822 (2,593, 3,051)	0.240
VO_2_ max; ml/kg/min	47.9 (45, 51)	45.4 (43, 48)	0.003^*^
Breathing frequency; per min	49 (45, 53)	44 (42, 47)	0.005^*^
Minute ventilation; liters	92.0 (84, 100)	94.7 (86, 103)	0.340
MVV; liters/min	109 (99, 108)	120 (111, 129)	<0.001^*^
RER	1.16 (1.13, 1.19)	1.19 (1.15, 1.22)	0.079
Height; cm	166 (163, 169)	169 (166, 172)	<0.001^*^
Weight; kg	57.7 (54, 61)	62.2 (59, 66)	<0.001^*^

*EILO, exercise induced laryngeal obstruction; VO_2_ max, maximal oxygen consumption; MVV, maximal minute ventilation; RER, respiratory exchange ratio; CI, confidence interval*.

a *All values are given as means (95% CI) at peak exercise*.

b *Paired sample t-test compare findings pre-operative vs. post-operative*,

* *p ≤ 0.05*.

### Subjective Symptom Scores

One patient did not complete the post-test questionnaire, for unknown reason. After surgery, perceived subjective symptoms improved in 38/44 (86%), were unchanged in 5/44 (11%), whereas 1/44 (2%) were unsure. The responses to the questions Q2, Q3 and Q4 improved significantly, and 25/43 (58%) patients (one did not answer this question) responded *nothing* or *a bit* to question Q4 (*When you are physically active, how much are you bothered by your breathing difficulties after surgery?)* (Table 3). Two patients reported more symptoms after surgery; one of whom had a post-operative complication.

**Table 3:** Symptom scores based on four questions from the 45 patients included from the EILO register at the Haukeland University Hospital, Bergen (Norway) who were surgically treated for EILO during 2013–2015.

**Question^a^**	**Pre-operative**	**Post-operative**	***p*-value^b^**
Q1. “Has your symptoms improved after the previous test?”: *n* (%)		Yes 38 (86.4)^†^	
		No 5 (11.1)^†^	
		Unsure 1 (2.3)^†^	
Q2. Rate your breathing problems (NRS-scale from 0 to 10): mean (95% CI)	7.4 (6.8, 8.1)	2.8 (1.9, 3.6)	<0.001^*^
Q3^a^. “I experience inspiratory breathing difficulty when I exercise?” (NRS 0–5): mean (95% CI)	4.2 (3.9, 4.6)	2.6 (2.1, 3.0)	<0.001^*^
Q4^a^. “How much are you bothered by your breathing difficulties” (NRS 0–5): mean (95% CI)	3.8 (3.5, 4.2)	1.7 (1.4, 2.1)	<0.001^*^

*EILO, exercise induced laryngeal obstruction; NRS, numeric rating scale (0–10); CI, confidence interval*.

† *One patient did not answer the question*.

a *Answer options: 1 = never, 2 = sometimes, 3 = Often, 4 = Almost every time, 5 = Always*.

b *p-Value from student paired t-test*.

* *p ≤ 0.05*.

### Laryngeal Findings During Exercise (CLE-Score) and Complications

**Figure 3:**
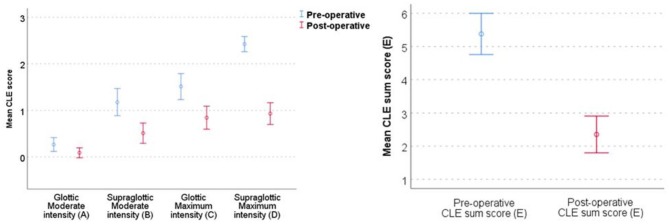
Mean CLE sub scores and sum score (E) with 95% CIs at glottic (A + C) and supraglottic (B + D) level, and at moderate and at maximum intensity before and after supraglottoplasty from 45 patients included from the EILO register at Haukeland University Hospital, Bergen (Norway) who were treated with supraglottoplasty during 2013–2015. CLE, continuous laryngoscopy exercise test; EILO, exercise induced laryngeal obstruction; CI, confidence interval. See Figure 1 for illustrations on CLE score (laryngeal obstruction scores).

**Figure 4:**
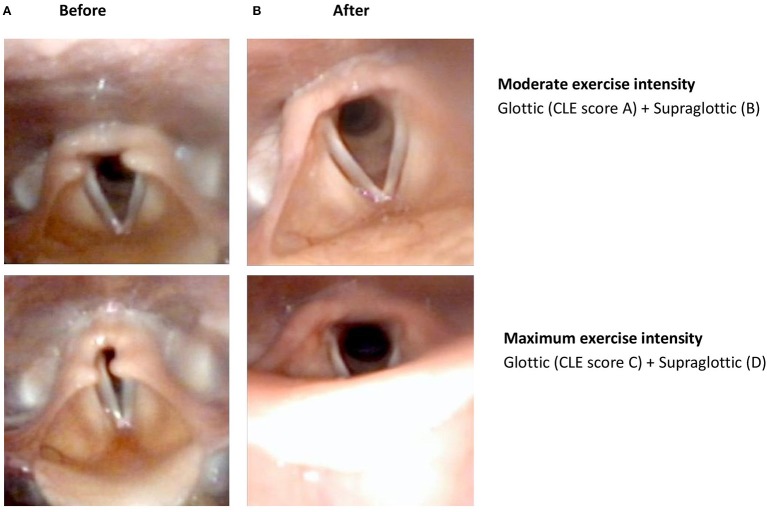
Laryngoscopic findings in a patient with exercise-induced laryngeal obstruction (EILO), both supraglottic and glottic obstruction during maximum exercise intensity before **(A)** endoscopic supraglottoplasty and open laryngeal inlet after **(B)** endoscopic supraglottoplasty. Regarding CLE-scores, see Figure 1 for illustrations.

Laryngoscopy at rest was normal in all subjects. All patients had significantly lower CLE-scores after surgery, with sum-score (E) significantly reduced from 5.38 to 2.36; most improvements explained by reduced supraglottic scores at maximum exercise (CLE D-score) (Table 4 and Figure 3). After surgery, 16/45 (36%) patients had no signs of laryngeal obstruction at maximum exercise intensity, i.e., CLE sum-score 0 or 1 (Figure 4). In 38/45 (84%) patients, CLE sum-score was reduced by 2 or more, reductions mainly occurring at the supraglottic level, as expected. However, in 21/45 also the glottic obstruction decreased at maximum intensity exercise, 10 of whom with a reduction of the glottic score of ≥2. In one patient, only the glottic obstruction improved, reducing CLE C-score (glottic score at maximum exercise) from 3 to 1. In two patients, the glottic obstruction worsened after surgery, but due to reduced supraglottic obstruction, the overall sum-score was reduced. One of these two had pre-operative glottic obstruction already at moderate intensity exercise, while the other was one of the two who experienced a post-operative complication.

**Table 4:** Continuous laryngoscopy exercise (CLE) scores from the 45 patients included from the EILO register at Haukeland University Hospital, Bergen (Norway) who were surgically treated for EILO during 2013–2015^a^.

**CLE conditions**	**Pre-operative**	**Post-operative**	**Pre-post change**	
	**Mean (SD)**	**Mean (SD)**	**Mean (SD)**	**95% CI**
**MODERATE INTENSITY**				
Glottic (CLE A)	0.27 (0.49)	0.09 (0.36)	0.18 (0.44)	(−0.08, 0.43)
Supraglottic (CLE B)	1.18 (0.96)	0.49 (0.73)	0.69 (0.73)	(0.43, 0.94)
**MAXIMUM INTENSITY**				
Glottic (CLE C)	1.51 (0.92)	0.84 (0.93)	0.67 (0.93)	(0.41, 0.92)
Supraglottic (CLE D)	2.42 (0.54)	0.93 (0.78)	1.49 (0.81)	(1.23, 1.74)
Sum score (CLE E)	5.38 (2.07)	2.36 (1.85)	3.02 (1.60)	(2.54, 3.50)^b^

*CLE A, glottic obstruction at moderate intensity; CLE B, supraglottic obstruction at moderate intensity; CLE C, glottic obstruction at maximum intensity; CLE D, supraglottic obstruction at maximum intensity; CLE E, sum-score (A + B + C + D), see Figure 1 for illustrations on CLE score; CI, confidence interval; SD, standard deviation*.

a In the final mixed linear regression model: CLE-score = constant + surgery + Intensity + location + surgery*Intensity + surgery*location, where all included effects were significant (p < 0.001).

b *Estimates from Student's paired t-test, p < 0.001*.

In the mixed linear regression model the three-way interaction was not significant (*p* = 0.231), neither was the two-way interaction between exercise intensity (moderate vs. maximum) and level of obstruction (glottic vs. supraglottic) (*p* = 0.232). The final model included interactions between pre- vs. post-operative and moderate vs. maximum exercise intensity, and pre- vs. post-operative and glottic vs. supraglottic obstruction (both *p*-values < 0.001). Thus, the CLE-score change after surgery was larger at maximum than at moderate exercise intensity, both glottic and supraglottic. Moreover, the CLE-score change after surgery was larger at the supraglottic than at the glottic level, regardless of exercise intensity.

Post-operative complications occurred in one of the included patients; a left vocal fold paresis that spontaneously resolved 3 years later. One of the 16 patients excluded due to missing pre-operative data, experienced post-operative scarring. This patient did not report post-operative breathing difficulties during daily activities, but persistent respiratory symptoms during exercise. This patient was offered a re-operation but declined. Hence, correct complication rate in our data was 3% (2/65).

### Conservative Treatment

Second-line conservative treatment had been attempted in only 15/45 patients since no gold standard for EILO-treatment exist. Surgery was offered as a second or third-line option due to persistent symptoms and supraglottic obstruction. Fourteen patients underwent 6 weeks of inspiratory muscle training, whereas one underwent speech therapy over several weeks. The conservative treatment improved symptom scores (Q2) by 1.42 (*p* = 0.027), but patients perceived improvements as insufficient and their CLE-scores did not alter.

## Discussion

In this retrospective review of patients treated with laser supraglottoplasty for EILO, 86% reported perceived subjective symptom improvement. The CLE-sum score decreased in all patients; as expected most evident at the supraglottic level, but notably, there were also significant improvements at the glottic level. Our supraglottic findings are comparable to findings in previous studies (9, 27, 28, 36), thus supporting that laser supraglottoplasty is an efficient treatment in severe supraglottic EILO. The favorable glottic response from surgery was a novel finding. The study underlines the heterogeneity of EILO, and the importance of thorough phenotyping before making a treatment plan. Complications were rare.

### Strength and Weakness

Our institution has performed more than 3,000 CLE-tests over a period of 15 years, with surgery performed in 3%. We have previously reported on 23 cases treated surgically for supraglottic EILO, not included in this study (27). This present case series is the largest published to date. It was a major strength that post-operative CLE-tests were used to assess outcome in all participants, contrasting some previous studies (9, 26–28, 39). Evaluations based on subjective patient reports are vulnerable to bias in both directions. There may be a positive placebo effect induced by the surgical procedure *per se*. On the other hand, patients can be disappointed that surgery treats only their EILO and not their physical capacity, as evident in this present study with virtually no changes in maximum oxygen consumption. Validity and reliability of the CLE scoring system varies somewhat between studies (28, 35, 40, 41); however, it seems reasonable to conclude that experience is a factor that influences these issues. In our study, four highly skilled raters scored the videos based on a consensus system, much like how CLE-tests are scored in everyday work. It has been suggested that familiarity with the test situation should somehow by itself improve the CLE-score; however, one third of our patients had tried additional conservative treatment before surgery with no significant reduction in CLE-scores with repeated tests. Similar maximum heart rate, minute volume and running distance at the pre-operative and post-operative CLE-test verified that the intensity was similar and that the laryngeal findings therefore could be compared.

Patients with EILO usually show normal supraglottic anatomy and laryngeal motion at rest and may show supraglottic appearance similar to laryngomalacia during exercise. Hence, Bent et al. labeled their observations “exercise induced laryngomalacia” (42). In order to avoid a mix between the adult type of laryngomalacia and EILO we chose to exclude four patients that had characteristics of laryngomalacia at rest (30). All four reported improvements of symptoms, and their mean CLE-sum scores improved substantially by 3.0 (data not shown).

An obvious weakness of this study was its retrospective design, with patients allocated to surgery based on clinical decisions, with no randomization, no blinding, and inconsistent follow-up times.

### Supraglottoplasty as Treatment for EILO

Smith et al. first described endoscopic laser supraglottoplasty for supraglottic EILO in 1995 (43). A systematic review based on 72 patients suggested that the procedure is safe and indicates a favorable clinical response (29). In some published studies, it is difficult to verify the specific technique used (29). Careful assessment of our post-operative CLE-test files suggests that in some cases, more of the redundant supraglottic tissue could have been removed. On the other hand, a careful approach is required when performing surgery in otherwise healthy adolescents, particularly as long-term effects from surgery are unknown, and as normal laryngeal function during high-intensity exercise is poorly described (44). Also, the natural course of EILO is unknown. Laryngeal structures are described to become more rigid with age, and thus perhaps more stable (45–47). Patients in rapid growth should probably primarily be offered conservative treatment (10). A follow-up study up to 5 years showed persistent laryngeal obstruction during exercise in patients treated conservatively, despite decreasing self-reported symptoms (27). Symptomatic improvement could have been related to reduced physical activity with age, challenging the idea that EILO improves spontaneously with age (48, 49).

### Post-operative Complications

Complications following supraglottoplasty for EILO have not been reported, possibly as few post-operative CLE-tests have been performed. Two studies have reported post-operative symptomatic complaints; one reporting dysphagia (9), and another breathing difficulties while exercising in cold air (27). In the present study, one participant experienced a self-limiting left vocal fold paresis, first believed to be due to luxation of the arytenoid cartilage inflicted by the intubation tube. However, extensive work-up revealed an Epstein-Barr viral infection and a large mediastinal thymus, possibly affecting the recurrent laryngeal nerve. Examination 3 years later showed normalized vocal fold movements and an open laryngeal inlet during exercise (CLE sum-score 0). Another patient excluded from participation as the pre-operative CLE-test could not be scored, had laryngeal scarring in the post-operative CLE-test. Previous laryngoscopies at rest revealed a laryngeal cyst, a condition that empirically is known to increase the risk for scarring. Patients with EILO are otherwise healthy young people, and therefore complication rates should be close to zero with potential gains carefully weighed against the risks (10, 26).

### Glottic Adduction

It has been stated that glottic closure is likely to be unresponsive to surgical treatment (16). However, we found significant improvements also at the glottic laryngeal level. We cannot easily explain this, but perhaps the Bernoulli's principle is involved; i.e., removal of redundant supraglottic tissue leads to a wider supraglottic entrance, possibly reducing airflow turbulence and thus less negative luminal pressure, and therefore less adduction of the vocal folds below. The recently published method for measuring trans-laryngeal resistance appears as a promising possibility for an objective numeric outcome that might shed light on these issues (50).

## Conclusion

Supraglottoplasty improves symptoms and reduces laryngeal obstruction in patients with a predominant supraglottic EILO, and appears safe and efficient in highly selected severe cases. Notably, supraglottoplasty might improve also glottic obstruction in patients with combined supraglottic and glottic obstruction. Our findings substantiate the heterogeneity of EILO, with phenotypes that require different treatment approaches. Risk of complications calls for careful selection of patients based on a multidisciplinary approach, with conservative treatment carefully tested prior to surgery. There is an urgent need for randomized studies and longer follow-up periods.

## Data Availability

The datasets generated for this study are available on request to the corresponding author.

## Ethics Statement

The studies involving human participants were reviewed and approved by Committee on Medical Research Ethics of Western Norway (REK number 2016/1898). Written informed consent to participate in this study was provided by the participants' legal guardian/next of kin.

## Author Contributions

AS and MH: substantial contributions to the conception of the work, data collection, and drafting the manuscript. MV: substantial contributions to the conception of the work, data collection, critically revising the work, and final approval of the version to be published. TA: substantial contributions to the conception of the work, critically revising the work, and final approval of the version to be published. IE and LS: substantial contributions to the conception of the work and data collection. J-HH substantial contributions to the conception of the work and critically revising the work and interpretation of data, final approval of the version to be published. TH: substantial contributions to the conception of the work and critically revising the work and interpretation of data, final approval of the version to be published. GE: substantial contributions to statistical analysis and interpretation of the work, final approval of the version to be published. OR and HC: substantial contributions to the conception of the work, data collection and critically revising the work and interpretation of data, final approval of the version to be published. All authors contributed to manuscript revision, read and approved the submitted version.

### Conflict of Interest Statement

Haukeland University Hospital owns parts of US patent No. 11/134551, protecting the commercial rights of the CLE-test. The authors declare that the research was conducted in the absence of any commercial or financial relationships that could be construed as a potential conflict of interest.

## Abbreviations

CLE-test: continuous laryngoscopy exercise test
EIB: exercise-induced asthma/bronchoconstriction
EIIS: exercise induced inspiratory symptoms
EILO: exercise induced laryngeal obstruction
FEV_1_: forced expiratory volume in first second
IMT: inspiratory muscle training
NRS: numeric rating scale
PCA-muscle: posterior cricoarytenoideus muscle
Pi_max_: maximal inspiratory mouth pressure
VAS: visual analog scale
VCD: vocal cord dysfunction.
